# Supplementary material for: Empirical methods for controlling false positives and estimating confidence in ChIP-Seq peaks
Source: BMC Bioinformatics. 2008 Dec 5;9:523. doi: 10.1186/1471-2105-9-523 (PMC2628906; doi:10.1186/1471-2105-9-523)
Supplement: Additional File 1 — A variety of html documents from the USeq web site detailing the available applications, their best usage, output file type descriptions, command line menus, etc. [file 1471-2105-9-523-S1.zip › USeqUserGuides/JavaDoc.zip!/JavaDoc/util/bio/seq/BinaryBooleanReaderWriter.html]

BinaryBooleanReaderWriter


|  |  |  |
| --- | --- | --- |
| **Overview** \| **Package** \| **Class** \| **Use** \| **Tree** \| **Deprecated** \| **Index** \| **Help** | |  |
| PREV CLASS   **NEXT CLASS** | **FRAMES**    **NO FRAMES**     **All Classes** |  |
| SUMMARY: NESTED \| FIELD \| CONSTR \| METHOD | DETAIL: FIELD \| CONSTR \| METHOD |  |


---


## util.bio.seq Class BinaryBooleanReaderWriter

```
java.lang.Object
  util.bio.seq.BinaryBooleanReaderWriter
```

---

``` public class BinaryBooleanReaderWriter extends java.lang.Object ```

Can read or write a compressed boolean[] by converting
each 8 booleans to a unique string and assigns them to byte.
Reads and writes the bytes.

---

**Constructor Summary**

`BinaryBooleanReaderWriter()`


| **Method Summary** | |
| --- | --- |
| `static boolean[]` | `appendToEight(boolean[] b)`             Copies and increases the size of the boolean[] to be divisible by 8. |
| `boolean[]` | `convert2Boolean(byte[] bytes)` |
| `byte[]` | `convert2Bytes(boolean[] booleans)`             Assumes the boolean[] is evenly divisable by 8. |
| `static java.lang.String` | `convert2String(boolean[] b, int startIndex, int stopIndex_Excluded)`             Converts a boolean[] to a 0=false,1=true String. |
| `boolean[]` | `getBooleans()` |
| `boolean[]` | `loadBinaryBooleanArray(java.io.File file)`             Loads the boolean[] booleans field from a compressed binary format. |
| `void` | `makeMaps()` |
| `boolean` | `saveBinaryBooleanArray(boolean[] toSave, java.io.File file)`             Saves a boolean[] in a compressed binary format. |

**Methods inherited from class java.lang.Object**

`equals, getClass, hashCode, notify, notifyAll, toString, wait, wait, wait`

**Constructor Detail**

### BinaryBooleanReaderWriter

```
public BinaryBooleanReaderWriter()
```


**Method Detail**

### saveBinaryBooleanArray

```
public boolean saveBinaryBooleanArray(boolean[] toSave,
                                      java.io.File file)
```

:   Saves a boolean[] in a compressed binary format.

    :   **Returns:**: boolean - true if successful.

---


### loadBinaryBooleanArray

```
public boolean[] loadBinaryBooleanArray(java.io.File file)
```

:   Loads the boolean[] booleans field from a compressed binary format.

    :   **Returns:**: boolean[] or null if problem is encountered.

---


### convert2String

```
public static java.lang.String convert2String(boolean[] b,
                                              int startIndex,
                                              int stopIndex_Excluded)
```

:   Converts a boolean[] to a 0=false,1=true String.

---


### convert2Boolean

```
public boolean[] convert2Boolean(byte[] bytes)
```

---


### convert2Bytes

```
public byte[] convert2Bytes(boolean[] booleans)
```

:   Assumes the boolean[] is evenly divisable by 8.

---


### appendToEight

```
public static boolean[] appendToEight(boolean[] b)
```

:   Copies and increases the size of the boolean[] to be divisible by 8.
    Returns the original array if it is already divisible by 8.

---


### makeMaps

```
public void makeMaps()
```

---


### getBooleans

```
public boolean[] getBooleans()
```


---


|  |  |  |
| --- | --- | --- |
| **Overview** \| **Package** \| **Class** \| **Use** \| **Tree** \| **Deprecated** \| **Index** \| **Help** | |  |
| PREV CLASS   **NEXT CLASS** | **FRAMES**    **NO FRAMES**     **All Classes** |  |
| SUMMARY: NESTED \| FIELD \| CONSTR \| METHOD | DETAIL: FIELD \| CONSTR \| METHOD |  |


---
